# Fibrous dysplasia mimicking skeletal involvement in Lymphoma

**DOI:** 10.1002/jha2.688

**Published:** 2023-04-25

**Authors:** Orla Gildea, Claire Burney, Julian Kabala

**Affiliations:** ^1^ Clinical Haematology University Hospitals Bristol and Weston NHS Foundation Trust Bristol UK; ^2^ Radiology University Hospital Bristol and Weston NHS Foundation Trust Bristol UK

1

We present the case of a 19‐year‐old male with nodular sclerosing classical Hodgkin lymphoma, who had an incidental finding of fibrous dysplasia of the bone. This caused 18‐F‐fluorodeoxyglucose (18‐F‐FDG) avidity on positron emission tomography‐computed tomography (PET‐CT) scan leading to incorrect initial staging.

This patient presented with a 4‐month history of enlarging cervical lymph nodes, without B symptoms. He had no prior medical history. His initial diagnostic 18‐F‐FDG PET‐CT demonstrated moderately avid left cervical and superior mediastinal lymphadenopathy and two partially sclerotic, partially lytic bone lesions. These were moderately avid, one in the proximal right humerus (SUV 6.1) and the second in the posterior distal humerus (SUV 8.1). There was no other avid lymphadenopathy (see Figure 1). He had a magnetic resonance imaging (MRI) scan and an orthopaedic opinion was sought; the lesions were felt likely to be skeletal involvement of his Hodgkin lymphoma. Therefore, his staging was 4A, with an international prognostic score of 2, and treatment was commenced following a RATHL approach starting with ABVD chemotherapy.

**FIGURE 1:**
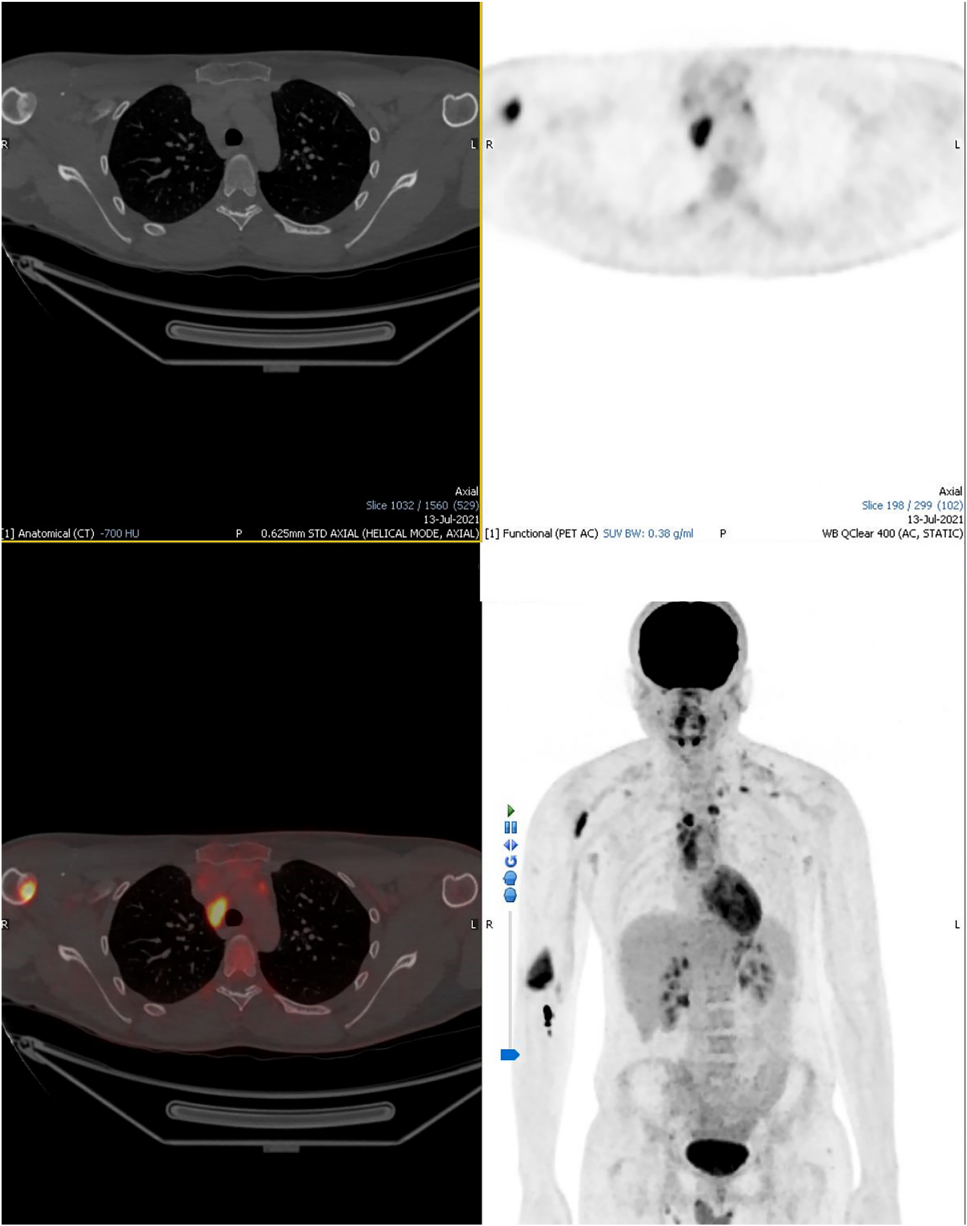
Initial staging PET ‐ CT demonstrating stage 4 disease.

The interim PET‐CT scan following cycle 2 of ABVD demonstrated a complete metabolic response within the cervical and mediastinal lymphadenopathy, but persistent avidity within the right humeral shaft lesions which were essentially unchanged in size with a mild reduction in 18‐F‐FDG avidity (see Figure 2). He did report some aching in the right arm. This discrepant radiological response prompted further investigation. The bone lesions were evaluated with a CT and MRI scan, and his case was discussed again with the orthopaedic team who at that point felt the radiological appearances were suggestive of fibrous dysplasia. He had a biopsy of the bone lesion and histological examination confirmed the diagnosis of fibrous dysplasia with no evidence of Hodgkin lymphoma or other primary bone malignancy. The patient's staging was therefore amended to stage 2A early favourable (ESR 4 mm/h at diagnosis), and treatment was changed to combined modality chemo‐radiotherapy. He had already completed 2 cycles of ABVD and so proceeded to receive 20 Gy in 10 fractions of consolidation radiotherapy. His end‐of‐treatment PET‐CT scan is awaited but clinically he remains in complete remission. He will require surveillance imaging of the bony lesions.

**FIGURE 2:**
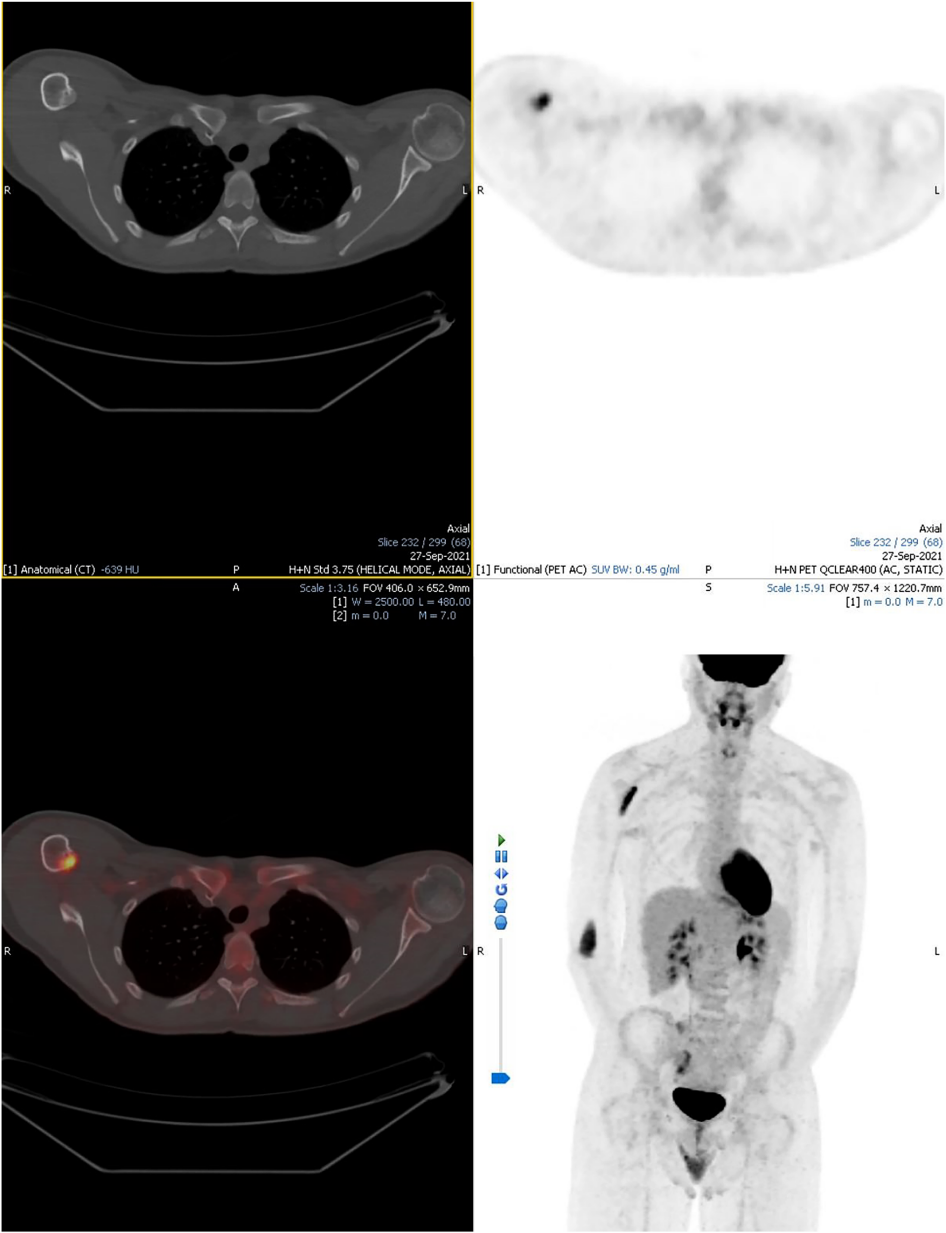
PET‐ CT following 2 cycles of ABVD.

Fibrous dysplasia of the bone (FD) is an uncommon genetic disorder causing benign fibroosseous bone lesions. It is diagnosed predominantly in children and young adults. It has a wide spectrum of presentations but is often asymptomatic and can be found incidentally. Malignant transformation can rarely occur. Radiological features can vary from classical ground glass lesions to cystic or sclerotic lesions. FD lesions can show a large variability in 18‐F‐FDG uptake on PET‐CT, and avidity in FD may change over time. There are several other case reports of FD mimicking skeletal involvement in lymphoma.

In conclusion, discrepant responses to treatment should be further investigated. Careful interpretation of 18‐F‐FDG avid bone lesions is required to avoid misdiagnosis or overtreatment. FD should be considered within the differential diagnosis, especially in younger patients.

## AUTHOR CONTRIBUTIONS

Orla Gildea: conception, literature review and writing—original draft preparation

Claire Burney: supervision and writing—review and editing

Julian Kabala: resources, visualisation

## CONFLICT OF INTEREST STATEMENT

The authors declare no conflict of interest.

## FUNDING INFORMATION

Self.

## ETHICS STATEMENT

N/A.

## PATIENT CONSENT STATEMENT

Uploaded.

## CLINICAL TRIAL REGISTRATION (INCLUDING TRIAL NUMBER)

N/A.

## Data Availability

Not applicable to this article as no new data were created or analysed.

